# Supplementary material for: Local heating of radiation belt electrons to ultra-relativistic energies
Source: Nat Commun. 2020 Sep 10;11:4533. doi: 10.1038/s41467-020-18053-z (PMC7483540; doi:10.1038/s41467-020-18053-z)
Supplement: Supplementary file 1 — Supplementary Information [file 41467_2020_18053_MOESM1_ESM.pdf]

Supplementary Material for: *Local Heating of Radiation Belt Electrons to Ultra-relativistic Energies*

Supplementary Note 1

Examination of how radial profiles of phase space density evolve allows for the differentiation between local acceleration and radial diffusion. Phase space density is presented in terms of the three invariants of particle motion, referred to as magnetic coordinates in the paper. The first invariant, μ, is associated with the particle gyration and is given by

$$\mu = \frac{p_\perp^2}{2m_0 B}, \tag{1}$$

where $p_\perp$ is the particle's momentum in the plane perpendicular to the magnetic field, B, and m_0 is the rest mass. As μ is proportional to the square of the perpendicular momentum, at the same location and pitch angle, higher energy electrons correspond to larger values of μ.

The second invariant, J, is given by

$$J = \oint p_\parallel ds, \tag{2}$$

where $p_\parallel$ is the momentum parallel to the field, integrated over the bounce path. When μ is conserved, the second invariant may also be expressed as K (as is done in this work)

$$K = \frac{J}{2\sqrt{2m_0\mu}} = \frac{1}{2}\oint \sqrt{B_m - B(s)}ds, \tag{3}$$

where B_m is the magnetic field strength at the particle's mirror point. The closer to the magnetic equator that a particle mirrors, the nearer to 90° the equatorial pitch angle, and the smaller the value of K.

The third and final adiabatic invariant is related to the particle drift motion, and is given by

$$\Phi = \oint \mathbf{B} \cdot d\mathbf{A}, \tag{4}$$

conserving the magnetic flux contained within a drift shell. A variation of the third adiabatic invariant, used in this study, is Roederer's L^* parameter [1]

$$L^* = \frac{2\pi M}{\Phi R_E}, \tag{5}$$

where M and R_E are the Earth's magnetic moment and radius respectively.

As can be seen from Supplementary Equations 1 and 3, μ and K correspond to electrons of different energies depending on the magnetic field strength, and therefore on location. In Fig 2 of the main text, the average electron energy at each L^* is shown for different values of μ and $K = 0.11\ R_E G^{1/2}$. The values of μ have been calculated with the locally measured magnetic field data from Van Allen Probes and K calculated using the TS07 external magnetic field model [2]. Fig 2 provides a good reference for the corresponding particle kinetic energies for the phase space density values in Figs 4 and 5.

Supplementary Method

The method used for calculating the phase space density from the Van Allen Probe observations is described in the Method section of the main paper. A model pitch angle distribution

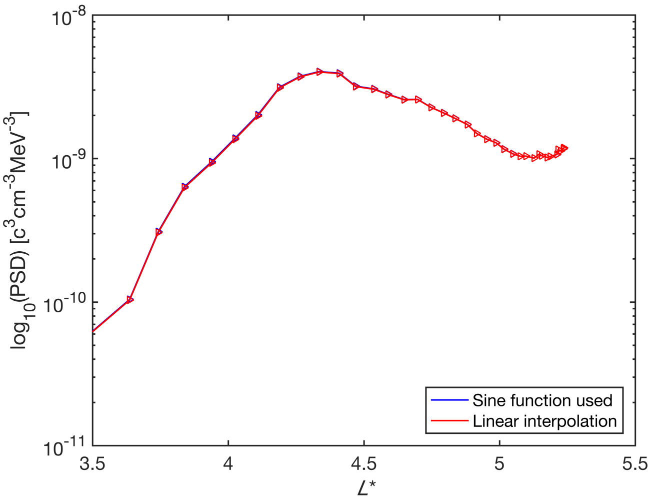

Supplementary Figure 1. Comparing two methods for phase space density (PSD) calculation. The phase space density profile at μ = 8,000 MeV G^{-1}, $K = 0.11\ R_E G^{1/2}$ achieved using a linear interpolation of the log of the phase space density (red line) is compared to that obtained using a $f(\alpha) = A\sin(\alpha) + C$ function between the two pitch angle channels immediately surrounding the target K value (blue line). The example pass is from Van Allen Probe A on October, 15 2012, 02:25 to 05:20 and arrow symbols indicate that the probe is on an outbound trajectory.

was not used when interpolating to the target K value. Instead, we linearly interpolate the logarithm of the phase space density to the target value of K. Alternatively, a functional form for the pitch angle distribution can be used to determine the interpolation. However, as shown in Supplementary Figure 1, using a $f(\alpha) = A\sin(\alpha) + C$ function between the two pitch angle channels immediately surrounding the target K has a very limited effect on the results.

Supplementary Note 2

In Supplementary Figure 2, phase space density data from both Van Allen Probes A and B are shown at eight values of μ and $K = 0.11\ R_E G^{1/2}$, for the period. Note that the same color scale is used for each μ to aid cross-comparison. Whilst the maximum level of phase space density decreases with increasing μ, Supplementary Figure 2 further demonstrates that the same general dynamics are seen across a broad energy range. Peaks in phase space density arise which persist for a number Van Allen Probes orbits.

In Fig 4 and 5, phase space density peaks were observed which grew with time for μ = 8,000 MeV G^{-1} and 10,000 MeV G^{-1} during both magnetic storms. By considering outbound and inbound passes of the Van Allen Probes, Supplementary Figure 3 further demonstrates that growing peaks were also observed at $K = 0.11\ R_E G^{1/2}$ for lower values of μ (3,000 MeV G^{-1} and 5,000 MeV G^{-1}).

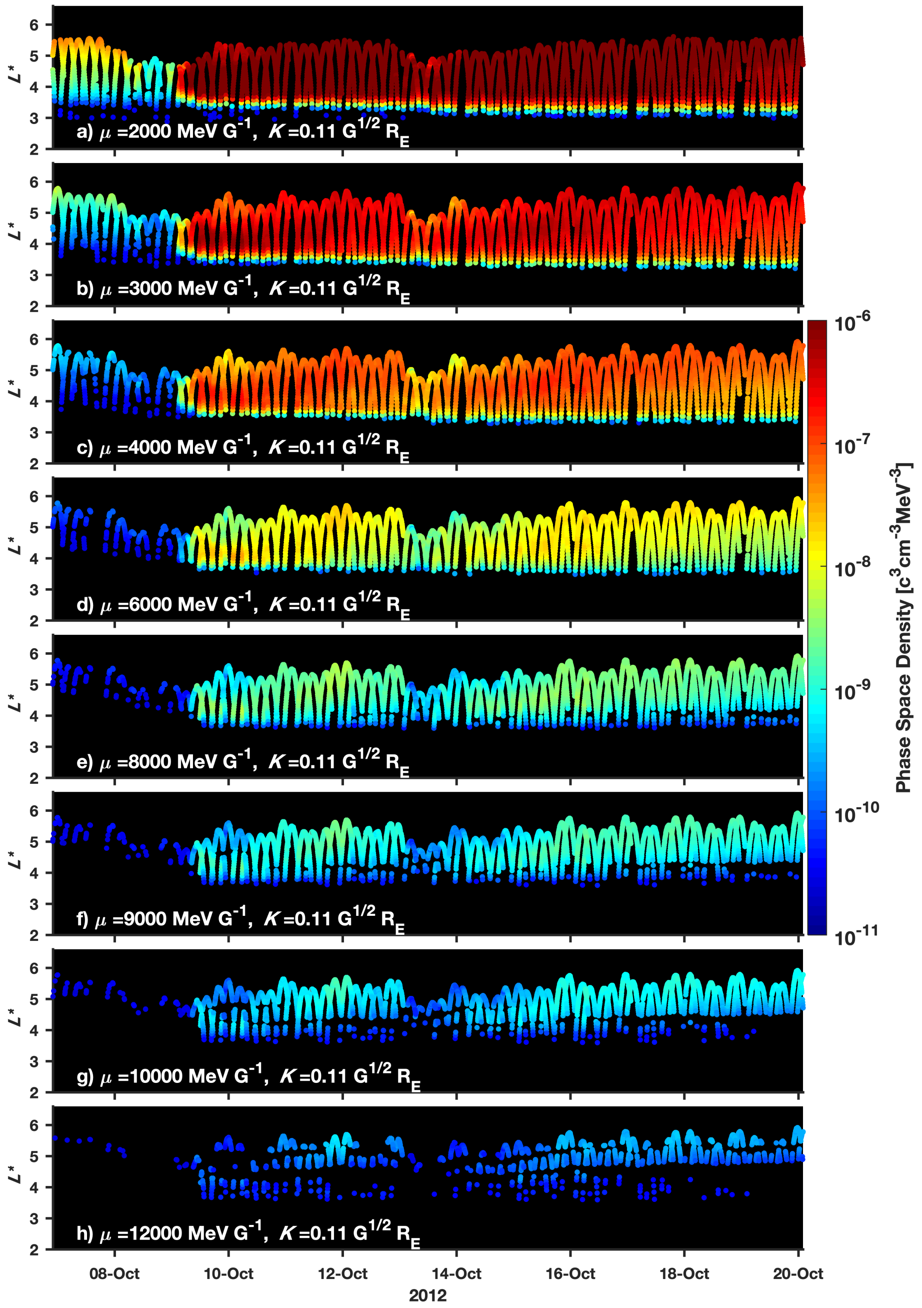

Supplementary Figure 2. Phase space density throughout the orbits of Van Allen Probes A and B. Values at $K = 0.11\ R_E G^{1/2}$ and eight different values of μ (panels a-h) are shown. Measurements below the background threshold of the energy channel [6] are not included here. The L^* and K values have been calculated using the TS07 magnetic field model [2], and μ calculated with the locally measured field.

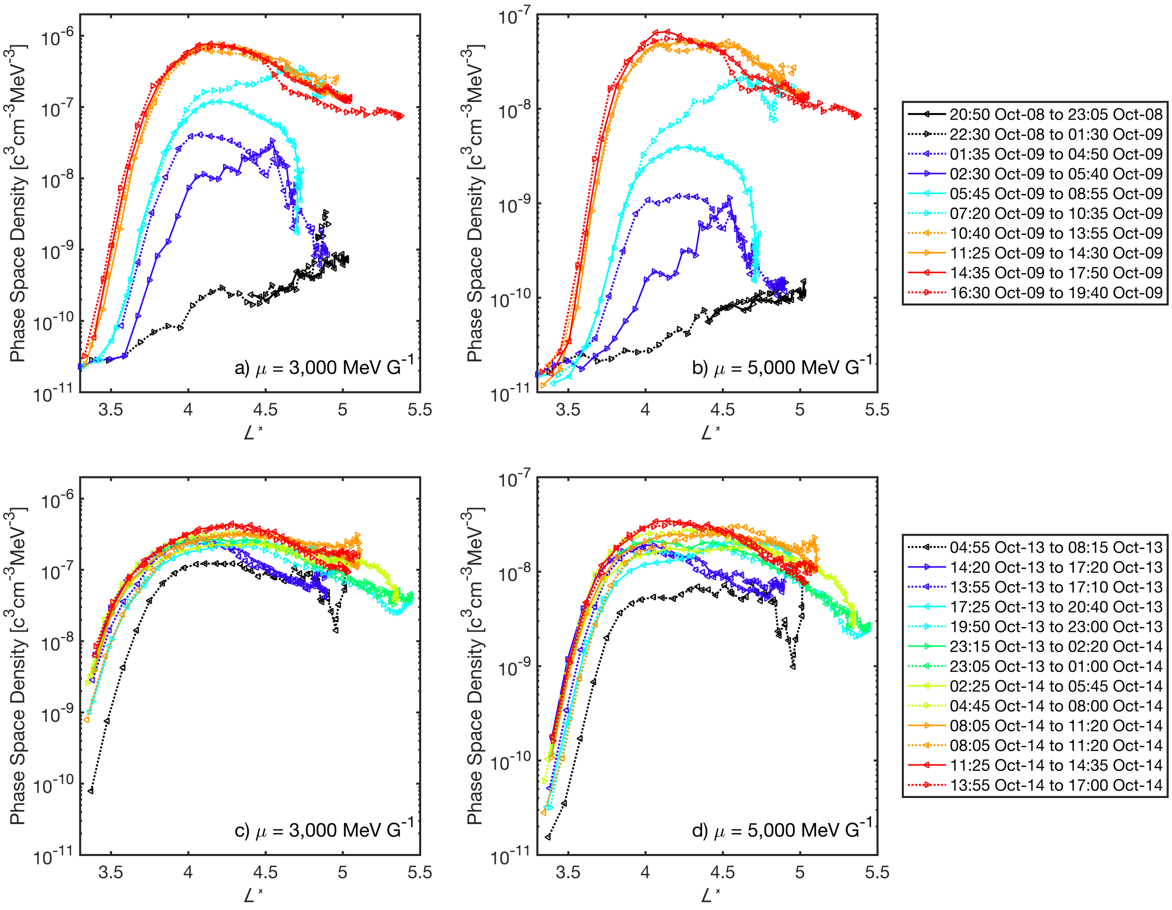

Supplementary Figure 3. Evolving phase space density - L^* profiles at lower μ. Panels (a) and (b) show the phase space density at μ = 3,000 MeV G^{-1} and 5,000 MeV G^{-1} respectively, during the first geomagnetic storm in October 2012. Panels (c) and (d) also show μ = 3,000 MeV G^{-1} and 5,000 MeV G^{-1}, but for the second storm event. All panels correspond to K = 0.11 $R_E G^{1/2}$. Van Allen Probe A is marked by solid lines and B by dotted lines as in Fig 5 of the main paper. Whether the satellite is on an inbound or outbound pass is marked by an arrow.

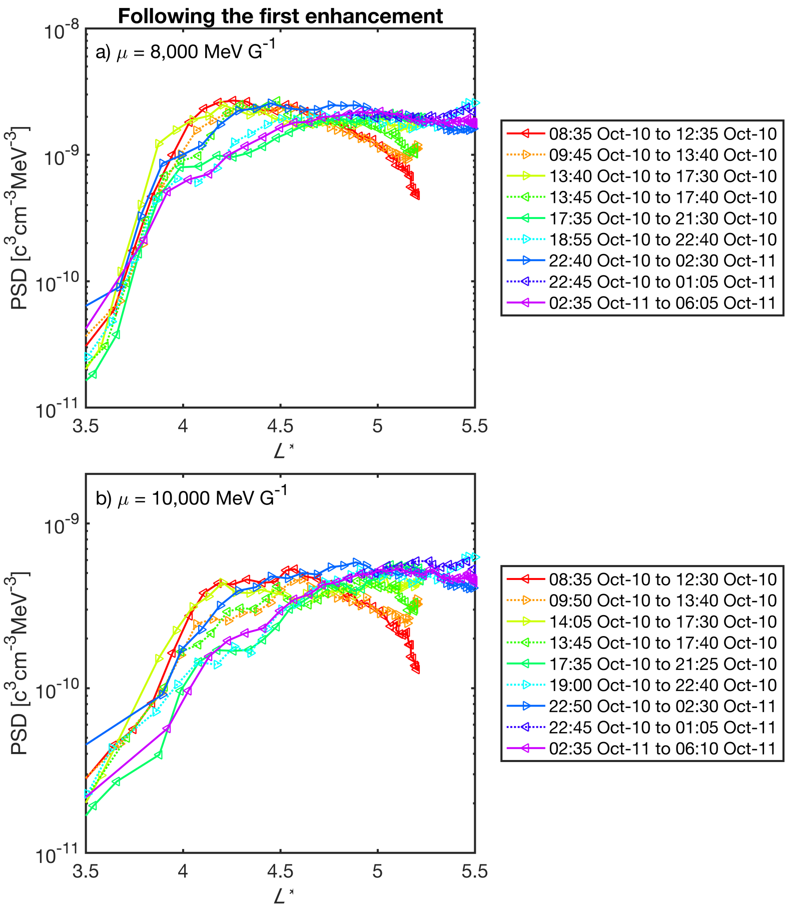

Supplementary Figure 4. Phase space density (PSD) - L^* profiles following the first enhancement. Panel (a) shows the results for $\mu = 8{,}000$ MeV G^{-1} and Panel (b) shows $\mu = 10{,}000$ MeV G^{-1}, both with $K = 0.11\ R_E G^{1/2}$. Van Allen Probe A values are plotted as solid lines and Van Allen Probe B measurements as dotted lines. Arrows indicate the direction of travel.

Supplementary Note 3

The phase space density profiles during the first 7 MeV enhancement are plotted in Fig 5a and b, showing the growth of a peak. Subsequent passes are shown in Supplementary Figure 4 to demonstrate that this peak later evolves into a plateau, likely due to outward radial diffusion as discussed in the main text.

Supplementary Note 4

The acceleration following the second storm continues over a longer period than for the first storm. Between the initial black pass in Fig 5c and d (main text) and those shown in dark blue, there is an additional inbound pass of Probe A and outbound pass of Probe B. During these passes, magnetopause shadowing was still prevalent, and while the phase space density values are similar to those shown black, the magnetic field was dynamic and L^* oscillates, introducing noise into the phase space density profile. Supplementary Figure 5 shows these additional passes in purple.

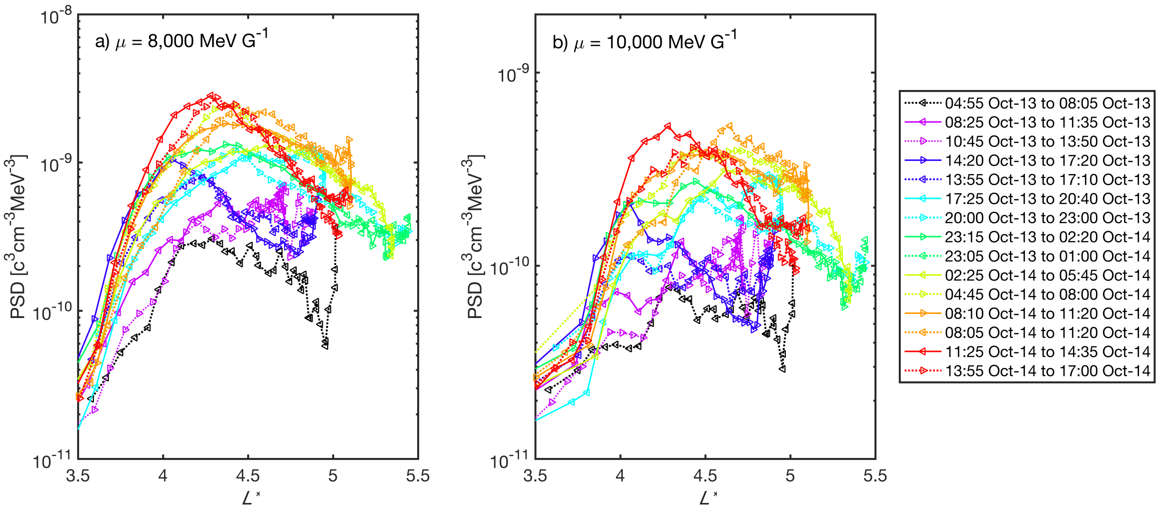

Supplementary Figure 5. Phase space density (PSD)-L^* profiles during the second enhancement event. Panel (a) shows the profiles for μ = 8,000 MeV G^{-1} and panel (b) shows profiles for μ = 10,000 MeV G^{-1}, both with $K = 0.11\ R_E G^{1/2}$. The figure takes a similar format to Fig 5c and d in the main paper but shows two additional passes during which the magnetosphere was highly disturbed and magnetopause shadowing was active. Van Allen Probe A values are plotted as solid lines and Van Allen Probe B measurements as dotted lines. Arrows indicate the direction of travel.

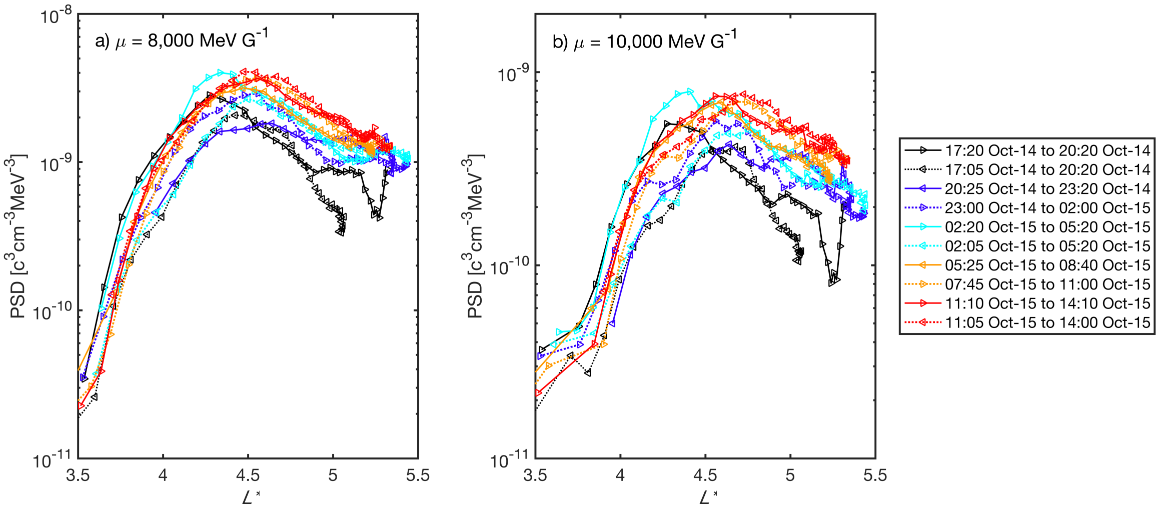

Supplementary Figure 6. Phase space density (PSD) - L^* profiles following the second enhancement. The profiles shown immediately proceed those in Fig 5c and d. Panel (a) shows the profiles for μ = 8,000 MeV G^{-1} and panel (b) shows profiles for μ = 10,000 MeV G^{-1}, both with $K = 0.11\ R_E G^{1/2}$. Van Allen Probe A values are plotted as solid lines and Van Allen Probe B measurements as dotted lines. Arrows indicate the direction of travel.

For both μ = 8,000 MeV G^{-1} and μ = 10,000 MeV G^{-1} the phase space density peak actually increases past the final profiles shown in Fig 5c and d (red pass). However, for clarity,

and to avoid over-cluttering the figure, the later passes were not shown in the main paper. Supplementary Figure 6 shows the phase space density profiles for the passes immediately following those shown in Fig 5c and d. A decrease is initially observed, with the phase space density evolving to more of a plateau form (dark blue pass probe A). Starting with the pass labeled 02:20 to 05:20 Oct-15 (light blue), the phase space density then grew once again, resulting in a peak centered at $L^* \sim 4.5$.

Supplementary Note 5

As Van Allen Probes A and B each only measure a single point in space at any one time, the phase space density - L^* profile is built up over a period of ~4.5 hours (half the orbit time). As a result, the phase space density for the L^* values towards the start of the pass originate hours earlier than those for the L^* values at the end of the pass. Here we show the timings for the Van Allen Probe passes during both events so that this time difference may be considered (Supplementary Figure 7 and Supplementary Figure 8).

The apogees of the two probes are separated by ~5 hour intervals. However, as can been seen from Supplementary Figure 7 and Supplementary Figure 8, the probes sample L^* values near the maximum L^* for some time, and the measurements within $0.2L^*$ of apogee are separated by ~2.5 hour intervals between probe A and B. A perceived growing peak from radial diffusion can be produced, in some circumstances, by a increase at the outer radiation belt L^* boundary to phase space densities higher than the observed peak in the heart of the belts, followed by a sudden decrease to phase space densities less than the observed peak [7]. To appear as a growing peak when seen by Van Allen Probes, both the increase and the decrease must occur between the Van Allen Probe passes, imposing a small time window for the process.

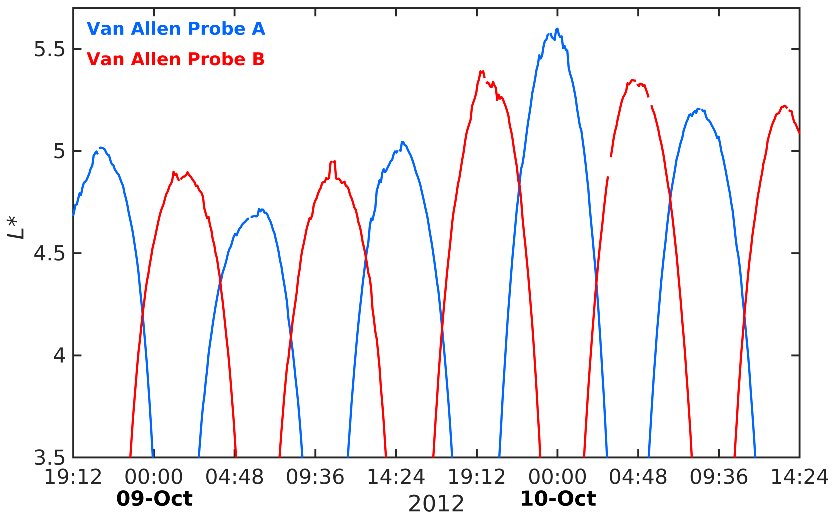

Supplementary Figure 7. Orbit times of the Van Allen Probes during the first storm event. Van Allen Probe A (blue line) and Van Allen Probe B (red line) L^* values shown correspond to $K = 0.11\ R_E G^{1/2}$ and have been calculated using the TS07 field model [2].

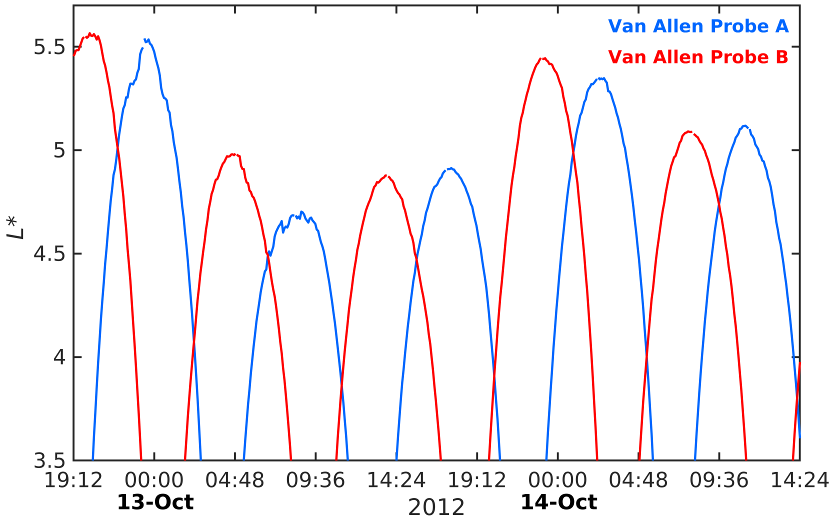

Supplementary Figure 8. Orbit times of the Van Allen Probes during the second storm event. Van Allen Probe A (blue line) and Van Allen Probe B (red line) L^* values shown correspond to $K = 0.11$ $R_E G^{1/2}$ and have been calculated using the TS07 field model [2].

Supplementary Note 6

The apogee of the Van Allen Probes is at a radius of 5.8 R_E, corresponding to L^* values frequently inside the last closed drift shell (L^*_{max}). For both of the two events of interest, we show the L^*_{max} value in Supplementary Figure 9 at $K = 0.11$ $R_E G^{1/2}$, calculated from the TS07 field model [2]. During the first storm in October 2012, L^*_{max} ~5.5 for the initial part of the enhancement. As electrons outside of L^*_{max} are not expected to be stably trapped, lost on a time frame of a drift period (minutes for MeV electrons), any source population for radial diffusion originates from $L^* < L^*_{max}$, imposing energy restrictions determined by the μ and K values. Alternatively, a source population could potentially be supplied by plasma sheet inflow or substorm injections. However, to achieve an enhancement of 7 MeV electrons at L^* ~4 while $L^*_{max} \sim 5.5$, Fig 2 indicates that we would require >4 MeV electrons to be directly supplied to the radiation belts.

NOAA's Geostationary Earth Operational Satellites (GOES) are located in geostationary orbit and sample L^* values around $L^* \sim 6$. Although the maximum L^* on the x-axis is $L^* = 5.7$, the trends shown in Fig 2 imply that for $L^* = 6$, $\mu = 10{,}000$ MeV G^{-1} and $\mu = 8{,}000$ MeV G^{-1} are likely to correspond to energies of ~4 MeV and ~3.5 MeV respectively. The relativistic electron flux data product from GOES, corrected for dead-time and proton contamination, is supplied for the >800 keV and >2 MeV channels. During the period of investigation, GOES 13, 14, and 15 were all operational and the flux for the two events are shown in Supplementary Figure 10 and Supplementary Figure 11. GOES measures a broad pitch angle range and the flux value provided is the average for the pitch angles sampled. L^* is a particle property and depends on the electron pitch angle [1]. As a result, the GOES flux at each time corresponds to a range of L^*. We therefore show the L^* values for five local pitch angles, spanning from 5° to 85°. It is likely that, often, the flux at the higher pitch angles will dominate the average. However, as is shown in Supplementary Figure 10, during some periods, the L^* coverage can span more than $0.5L^*$. When the higher pitch angles correspond to significantly higher L^*, flux measurements may be more representative of lower pitch angles.

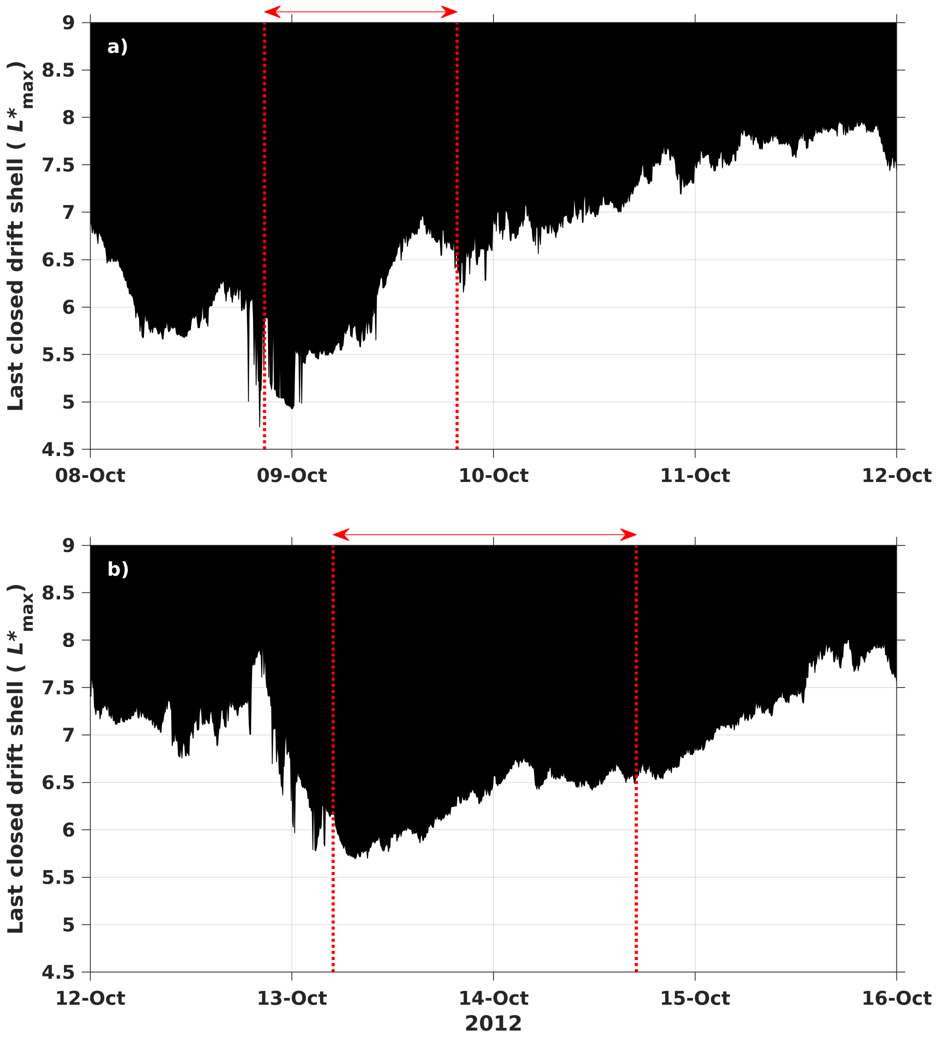

Supplementary Figure 9. The last closed drift shell, L^*_{max}, values. The L^*_{max} values correspond to K = 0.11 $R_E G^{1/2}$ and the time evolution is shown for the first storm (panel a) and second storm (panel b) as calculated using the TS07 field model [2]. Red arrows and dotted lines highlight the time range of the phase space density-L^* profiles in Fig 5.

Although the electron energies are lower than required for the observed 7 MeV enhancement, GOES data still offers insight into the electron dynamics outside of the region observed by Van Allen Probes. For the first event, shown in Supplementary Figure 10, the >2 MeV flux from GOES-13 increases from background levels during October 9. Two flux increases are seen, corresponding to reductions in L^* for all pitch angles. An extended 'bump' then appears while $L^*(\alpha{=}85^o)$ gradually reduced, sampling deeper into the outer radiation belt, after which, a flux decrease is observed as $L^*(\alpha{=}85^o)$ increases to L^* >6.5.

Other than the changes introduced by variations in L^*, we do not see evidence for rapid >2 MeV flux enhancements occurring between the Van Allen Probe passes, instead observing a gradual increase which maximises at the end of the period shown. GOES-14 observes similar results to GOES-13. Lower values of L^* are sampled by GOES-15 which, at one point, measures as low as L^* ~4.8. The GOES-15 flux is therefore higher than GOES-13 and GOES-14, but

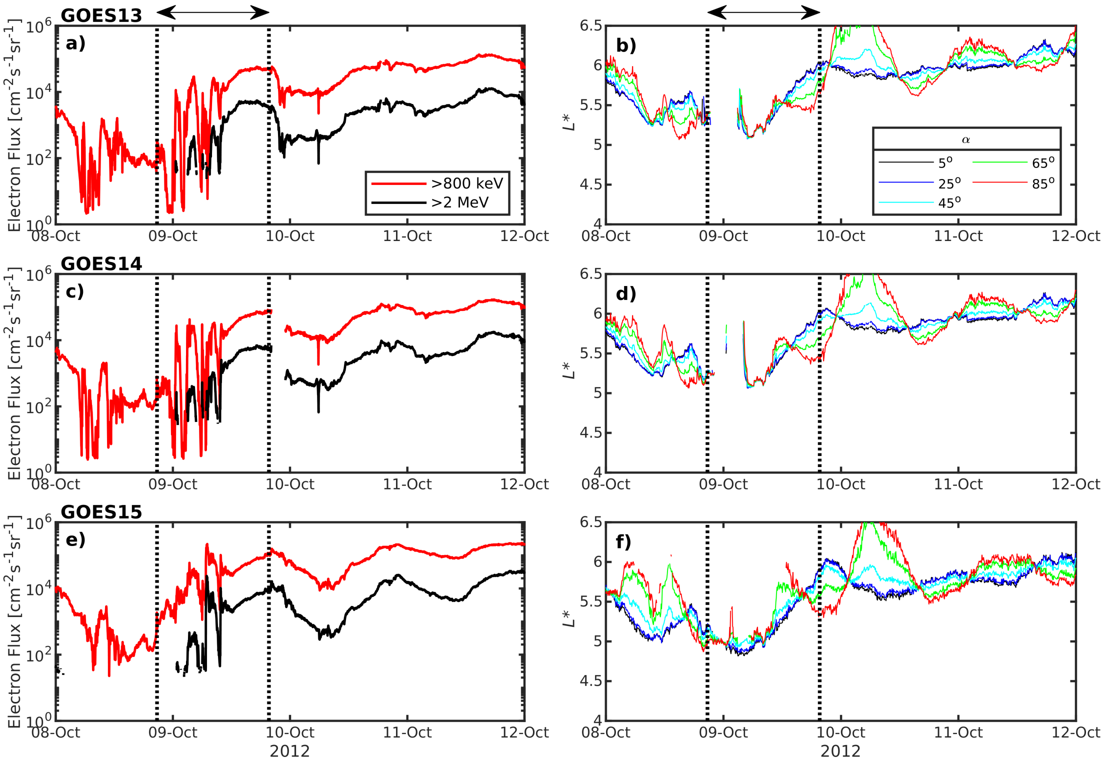

Supplementary Figure 10. Electron flux and L^* data from GOES satellites during the first event. The electron flux data from GOES 13, 14, and 15 during the first storm is shown in panels (a), (c), and (e) respectively. In each of these panels, red lines correspond to the >800 keV electron flux and black to the >2 MeV electron flux. GOES provides an integral electron flux measurement averaged over a broad range of local pitch angles. We therefore provide the L^* value for five local pitch angles, α, at each spacecraft in panels (b), (d), and (f) to give an idea of the L^* coverage of the measurement. All L^* values are calculated using the TS07 field model [2], consistent with the Van Allen Probes values shown in the main text. The arrows and dotted lines highlight the time-range of the phase space density-L^* profiles in Fig 5a and b.

again, the changes in the flux level can be attributed to changes in the L^* dynamics and we do not see evidence for rapid flux enhancements.

GOES data is also shown for the second storm in Supplementary Figure 10 and rapid enhancements are not observed between the Van Allen Probe orbits. Supplementary Figure 9 shows that L^*_{max} ranges between $L^* = 6$ and $L^* = 6.5$ for much of the highlighted period (arrow). There are two rapid drops in the flux observed by the GOES satellites. On both occasions the $L^*(\alpha{=}85^o)$ value was around $L^* = 5.5$ - 6, while L^*_{max} varied about $L^* = 6$. We therefore suggest that, on both occasions, these drop outs were the result of magnetopause shadowing effects [8].

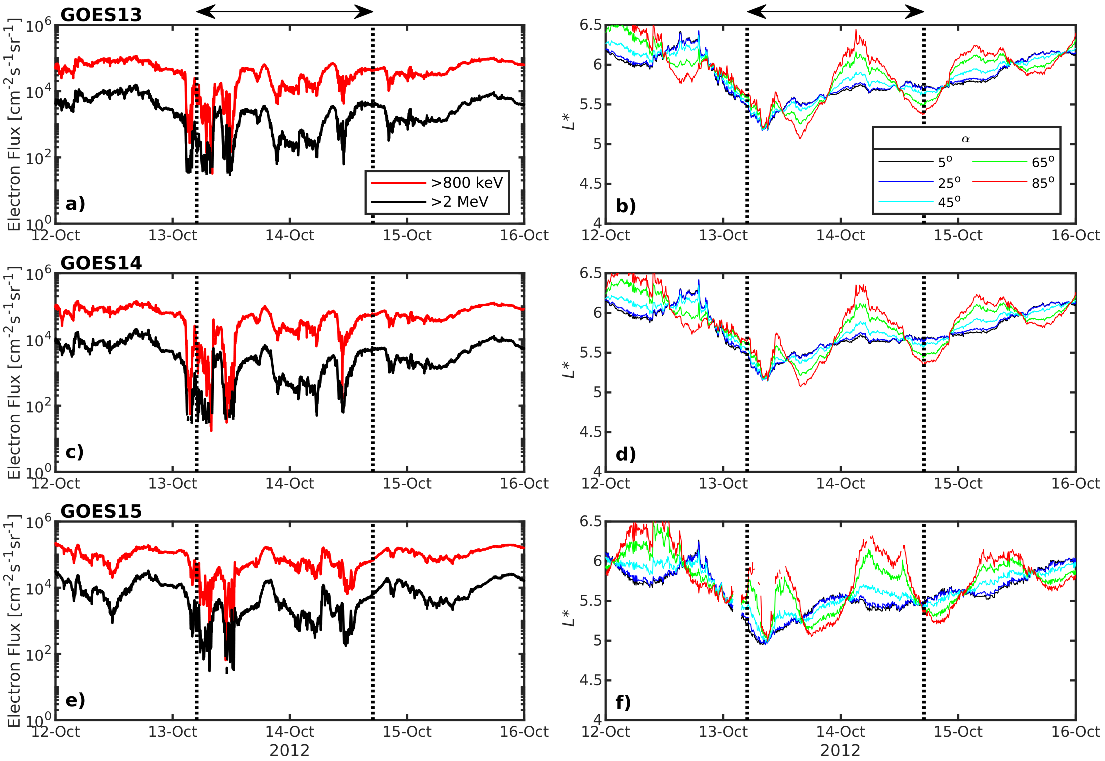

Supplementary Figure 11. Electron flux and L^* data from GOES satellites during the second event. This figure takes the same format as Supplementary Figure 10, here the >800 keV and >2 MeV electron flux measured by the three active GOES satellites during the second storm event are shown (panels a, c, and e) alongside the corresponding L^* values for five local pitch angles, α (panels b, d, and f). The arrow highlights the time-range of the phase space density-L^* profiles in Fig 5c and d.

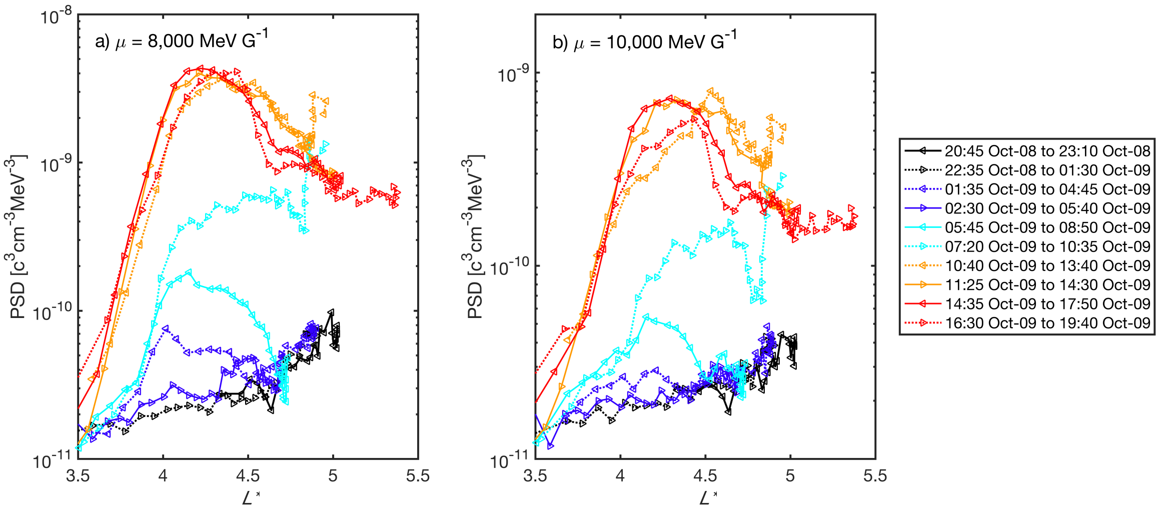

Supplementary Figure 12. Phase space density (PSD)-L^* profiles during the first storm over larger L^*. Panel (a) shows the results for μ = 8,000 MeV G^{-1} and panel (b) for μ = 10,000 MeV G^{-1}. For both panels, $K = 0.11\ R_E G^{1/2}$. Here we show a larger L^* range, which RBSP A and B do not sample for the majority of the passes, and the rapid enhancement at L^* ~4.8, which is likely due to errors in the magnetic field model. Van Allen Probe A values are plotted as solid lines and Van Allen Probe B measurements as dotted lines. Arrows indicate the direction of travel.

Supplementary Note 7

At $L^* \sim 4.8$, the outbound and the following inbound pass of Van Allen Probe B, between 10:00 and 11:00 on October 9 (see dotted blue and orange passes, Supplementary Figure 12), show a sharp change in phase space density for both μ = 8,000 MeV G^{-1} and μ = 10,000 MeV G^{-1}. Here we explore further the origin of this change.

Supplementary Figure 13 shows the local pitch angle, α, corresponding to $K = 0.11\ R_E G^{1/2}$ for each L^* during the outbound (green) and inbound (blue) sectors of the Van Allen Probe B orbit. The labels of the local pitch angle bins are also plotted as a reference and we see that, for $L^* \gtrsim$4.5 we interpolate between the 47.6^o and 37.1^o α bins. Over the L^* range where the rapid change in phase space density is observed, the α shows a sharp increase from the general trend for the outbound pass and a decrease for the inbound.

We also consider the corresponding energy for $K = 0.11\ R_E G^{1/2}$ at μ = 8,000 MeV G^{-1} (orange) and 10,000 MeV G^{-1} (blue), in Supplementary Figure 14. Again, for $L^* \gtrsim 4.8$, the profiles show departures from the general trend. On the outbound pass we see the corresponding energies fluctuate up and down, while on the inbound pass, a sharp decrease is observed.

As Supplementary Figure 13 and Supplementary Figure 14 do not show phase space density measurements, yet still show sharp changes over the same L^* range, we suggest that, at least in part, the phase space density increase seen at L^* ~4.8 is due to the magnetic field model. The L^* values for the two passes are shown in Supplementary Figure 15 for a range of local pitch angles. A sharp increase in L^* appears as the spacecraft approaches apogee, manifesting as a 'bump'. Very little L^* variation with pitch angle is seen for the outgoing pass. For reference, Supplementary Figure 16 displays the orbits of the two probes, indicating that probe B was in the dawn sector for the period.

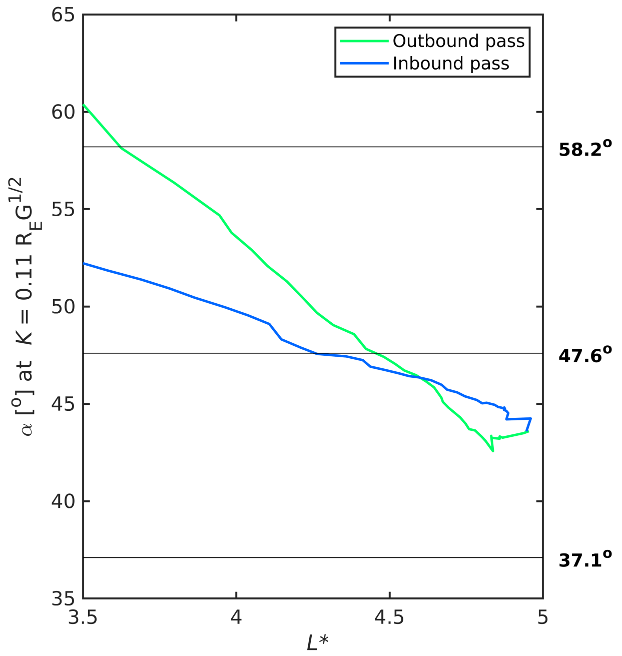

Supplementary Figure 13. Local pitch angle, α, at each L^* corresponding to $K = 0.11\ R_E G^{1/2}$. Here we show the values for the outbound (green line) and inbound (blue line) passes of Van Allen Probe B, surrounding 10:35:00 on October 9. The pitch angle values of the Van Allen Probes data are labelled as black horizontal lines for reference. L^* values and K values were calculated using the TS07 field model [2].

The ratio between the magnetic field from the TS07 field model and the observed magnetic field is shown on a log scale in Supplementary Figure 17 for both the outbound (green) and inbound pass (blue). A ratio of 1 is marked. Towards the end of the outbound pass the ratio fluctuates suggesting that the magnetic field model is not fully capturing the observed changes. Similar behaviour is seen for the inbound leg. Supplementary Figure 18 shows that the B_z component of the Earth's magnetic field increases and then decreases slightly between 10:00 and 11:00 UTC on the October 9th.

The electron flux measurements in the 3.4, 4.2, and 5.2 MeV energy channels of REPT are shown in Supplementary Figure 19 for the outbound and inbound passes surrounding 10:35 on October 9, 2012. We note that, at 3.4 MeV and 4.2 MeV, towards the end of the outbound pass, we see the electron flux increase for $\alpha \leq 58.2^o$. At higher pitch angles this increase is not observed. Ultimately, the pitch angle distribution inverts, with the flux level at $\alpha = 26.5^o$ surpassing that at 90°. As Supplementary Figure 13 shows that $K = 0.11\ R_E G^{1/2}$ corresponds to local pitch angles between 47.6^o and 37.1^o, the flux increase at $\alpha \leq 58.2^o$ may also contribute to the observed phase space density enhancement at $L^* \gtrsim 4.8$.

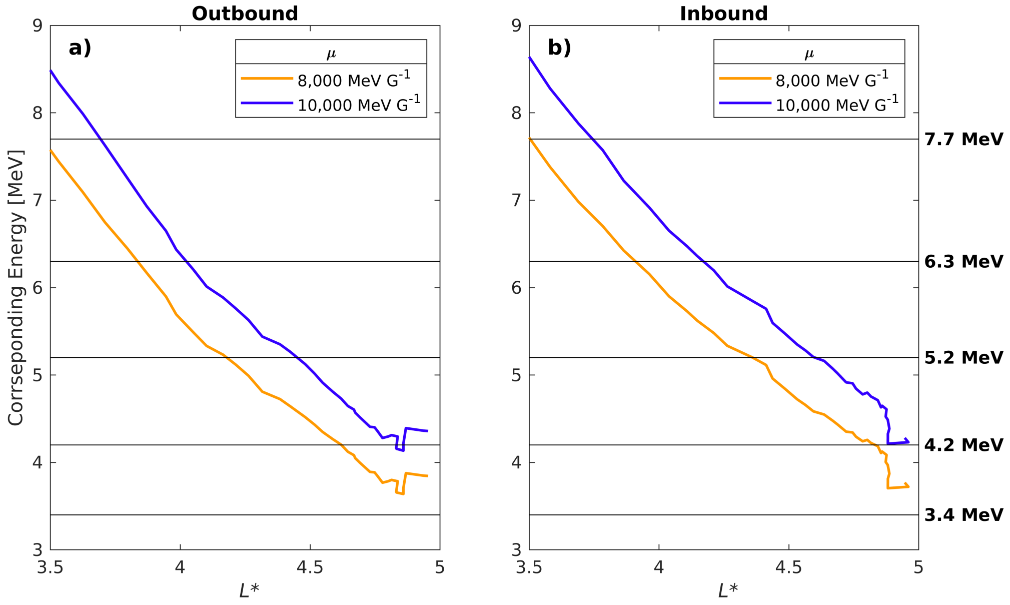

Supplementary Figure 14. The electron energy at each L^*. The energies shown correspond to $K = 0.11\ R_E G^{1/2}$ and either μ = 8,000 MeV G^{-1} (orange lines) or 10,000 MeV G^{-1} (blue lines), during the outbound (panel a) and inbound (panel b) passes of Van Allen Probe B surrounding 10:35:00 on October 9. The energy channels of the REPT instrument are labelled as black horizontal lines. L^* values and K values were calculated using the TS07 field model [2], while μ values utilise the locally measured field.

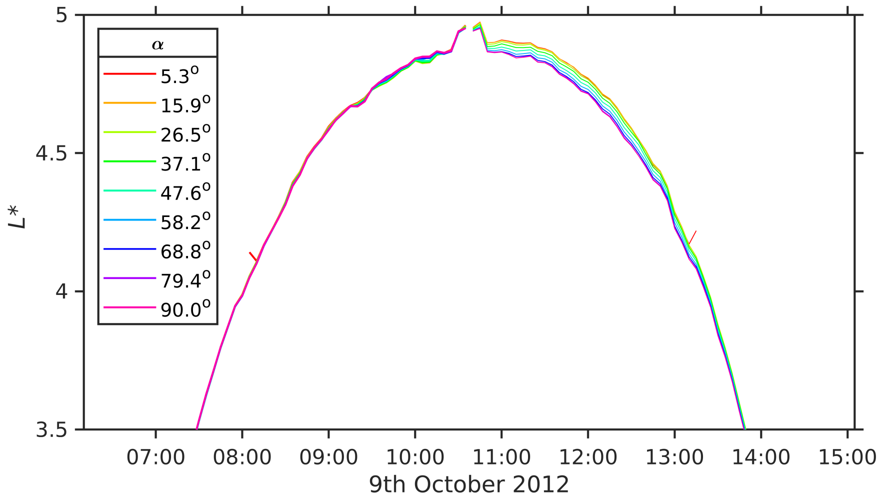

Supplementary Figure 15. L^* values for Van Allen Probe B, during 07:00 to 14:00 October 9. We show L^* for a range of local pitch angles, α.

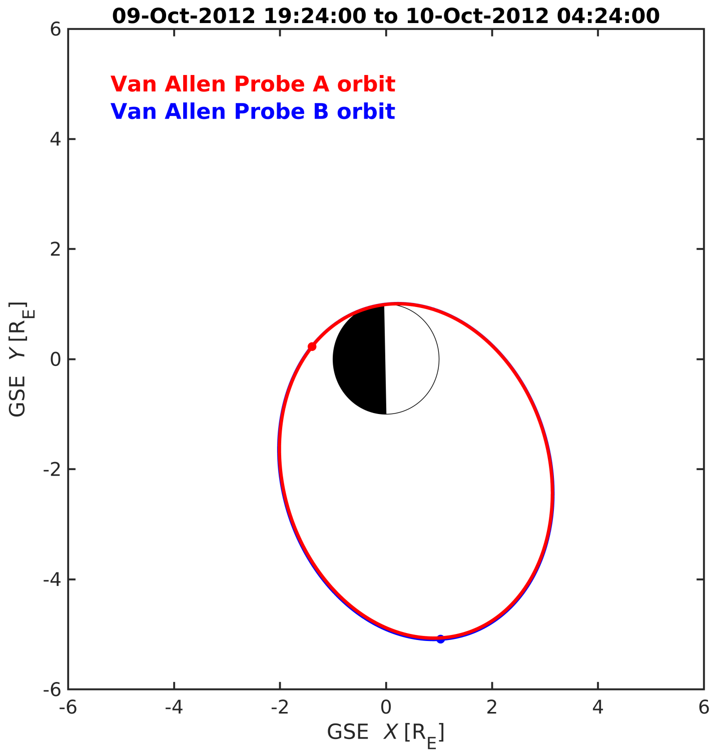

Supplementary Figure 16. Orbits of Van Allen Probe A (red) and B (blue) in GSE coordinates. Earth is shown for reference with the Sun-facing side marked in white and the night-side marked in black. The sample orbits shown are from the time period of 19:24:00 October 9 to 04:24:00 October 10. For the period considered in this study, the Van Allen Probes sample the dawn sector.

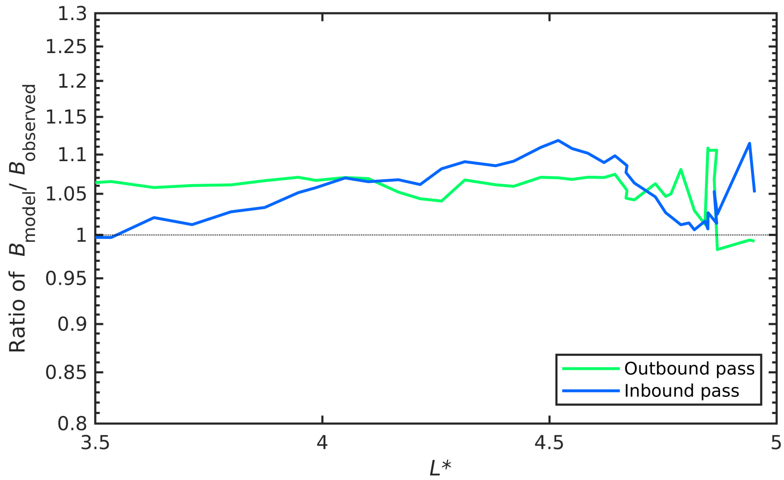

Supplementary Figure 17. Ratio indicating field model performance. The ratio of the TS07 magnetic field, B_{model}, to the observed magnetic field from Van Allen Probe B measurements, B_{observed} during the outbound (green line) and inbound (blue line) passes surrounding 10:35:00 on October 9. A ratio of 1 is marked for reference.

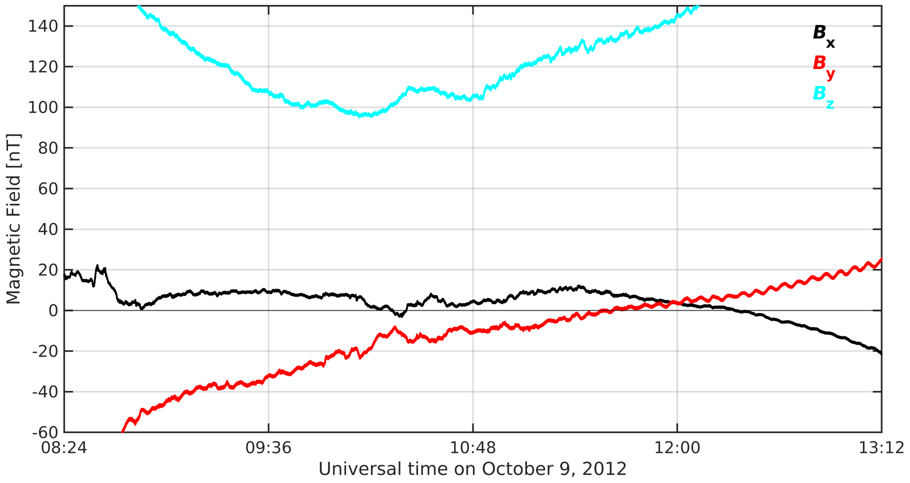

Supplementary Figure 18. Components of the measured magnetic field. The B_x (black), B_y (red), and B_z (cyan) components of the magnetic field measured by Electric and Magnetic Field Instrument Suite and Integrated Science (EMFISIS) [9] on Van Allen Probe B (in GEI coordinates) for 08:24 to 13:12 on October 9, 2012.

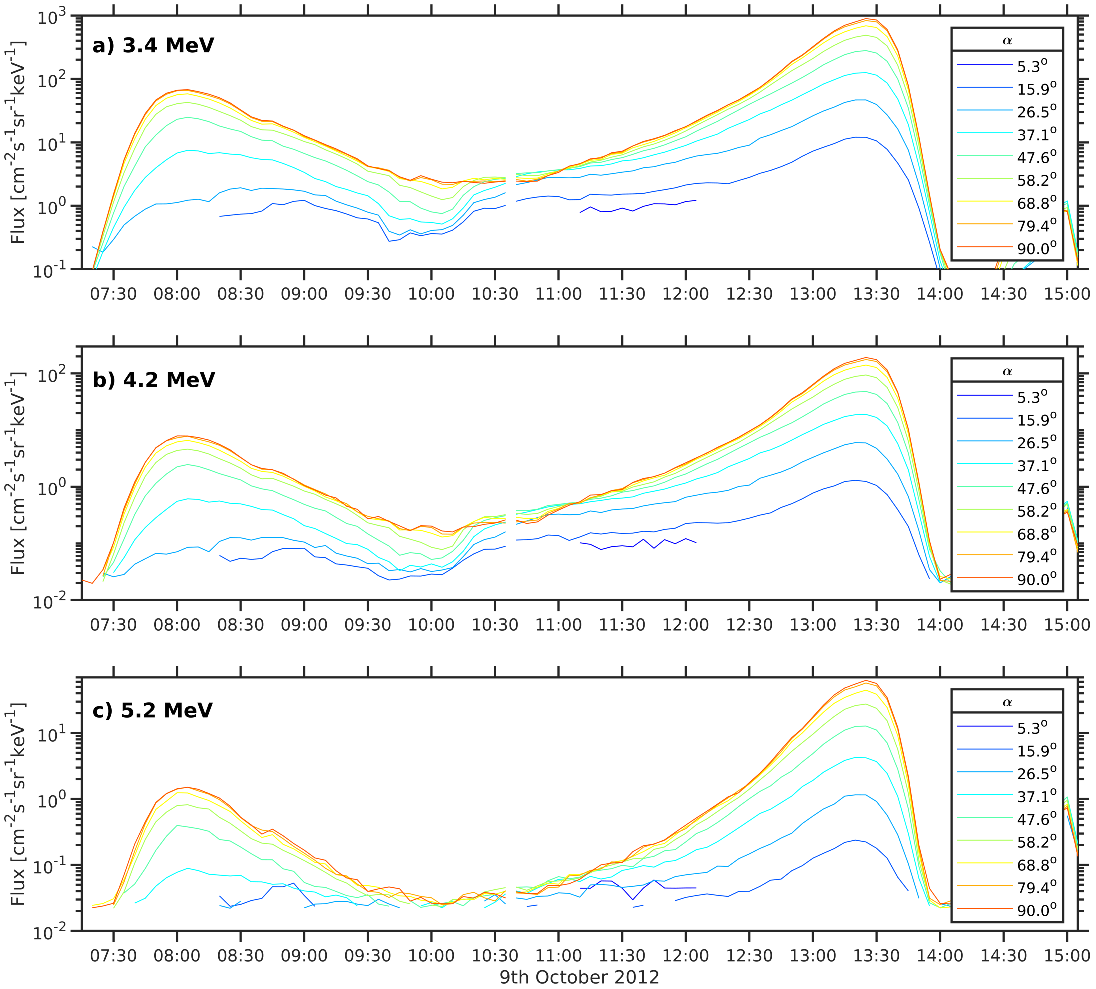

Supplementary Figure 19. Electron flux values measured along the orbit of Van Allen Probe B. The electron flux from the REPT instrument is shown at energies of 3.4 MeV (panel a), 4.2 MeV (panel b), and 5.2 MeV (panel c) for a range of local pitch angles, α, during the outbound and inbound pass of Van Allen Probe B surrounding 10:35:00, October 9.

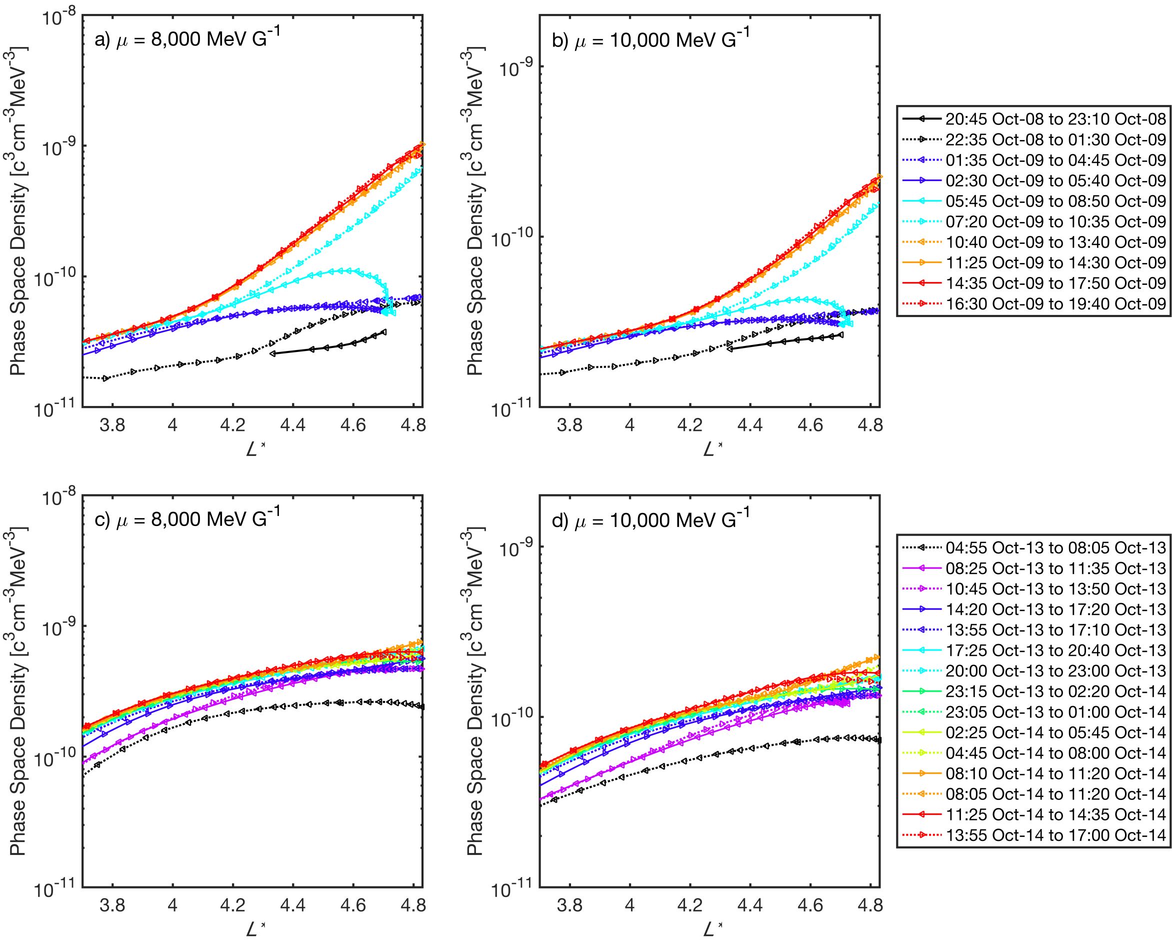

Supplementary Figure 20. Phase Space Density - L^* profiles simulated by VERB-1D. Phase space density profiles from VERB-1D runs are shown corresponding to the L^* and times of the Van Allen Probe A and B passes shown in Fig 5 in the main text. Results from the first storm event are shown for μ = 8,000 MeV G^{-1} and and μ = 10,000 MeV G^{-1} in panels (a) and (b) respectively. Panels (c) and (d) show the phase space density at μ = 8,000 MeV G^{-1} and and μ = 10,000 MeV G^{-1} during the second storm. All results correspond to $K = 0.11\ R_E G^{1/2}$. The figure takes the same format as Fig 5 in the main text. Phase Space Density values corresponding to the L* values and times of Van Allen Probe A are plotted as solid lines and Van Allen Probe B as dotted lines. Arrows indicate the direction of travel for each probe.

To explore how the phase space density profiles would evolve if radial diffusion was the sole process acting for ultra-relativistic energies, we use the 1-D setup of the Versatile Electron Radiation Belt (VERB) model [10–12] to solve the following equation:

$$\frac{\partial f}{\partial t} = L^2 \frac{\partial}{\partial L^*}\bigg|_{\mu,J} \left(L^{-2} D_{LL} \frac{\partial f}{\partial L^*}\bigg|_{\mu,J} \right) \tag{6}$$

where f is the phase space density, L is L^*, t denotes time, and D_{LL} is the radial diffusion coefficient. Here we have used the Brautigam and Albert [13] Kp driven parameterisation for D_{LL}, along with the measured Kp values for the period. Notice that we have neglected loss due to wave particle interactions altogether, assuming that for the two events, the plasmasphere was so eroded that hiss waves were not active over the L^* range considered [14] and that scattering

due to chorus interactions is minimal for these energies [15]. We use an outer boundary condition set by the phase space density at $L^* = 5$ (if the pass did not reach $L^* = 5$ then we used the closest available measurement and apply it at $L^* = 5$). Each of the two storms were run individually for a 1.4 day period, with initial conditions set by the phase space density observations.

Supplementary Figure 20 shows the outputs of the VERB-1D runs, interpolated to the same times and L^* values as the Van Allen Probe passes in Fig 5 of the main text. For both of the two events, the VERB-1D results show a phase space density around $L^* = 4$ that is considerably lower than observations. We also note that a very different structure is produced by VERB-1D to that observed, suggesting that, alone, radial diffusion is insufficient to explain the observed enhancements at these energies.

The increase seen in the phase space density at $L^* \gtrsim 4.8$ of pass 07:20 to 10:35 Oct-09 and pass 10:40 to 13:40 of Van Allen Probe B, discussed above in Supplementary Note 7, is included the outer boundary condition for the first event. It would therefore appear that, even with this increase, radial diffusion does not explain the evolution. The VERB-1D runs therefore support our interpretation that local acceleration was largely responsible for the growing phase space density peak observed.

Supplementary Note 8

Considering different values of K, as well as different values of μ, further helps determine the mechanism for relativistic electron heating. By pitch angle diffusion to lower values of α, a population originally enhanced by radial diffusion at low K may appear as a localized peak at higher K. Previously, we considered only $K = 0.11\ R_E G^{1/2}$, consistent with the work of previous authors [6, 16]. Smaller values of K correspond to electron populations with pitch angles closer to 90°, for which the enhancement may manifest differently. To explore this, we also present the phase space density at $K = 0.05\ R_E G^{1/2}$ (Supplementary Figure 21) where a stronger enhancement is seen on 9 October for $\mu = 12{,}000$ MeV G^{-1} than in Supplementary Figure 2. However, it is important to note that for a given μ, the electron energy at each L^* also changes with K. Supplementary Figure 22 uses the same format as Fig 2 and shows the average energy at each L^* for 10 values of μ during the 6-20 October 2013 period. At $L^* = 4$, $K = 0.05\ R_E G^{1/2}$, $\mu = \sim 12{,}000$ MeV G^{-1} electrons correspond to 7 MeV, while at $K = 0.11$ $R_E G^{1/2}$, only a μ value of $\sim$9,000 MeV G^{-1} is required to reach the same energy.

The two storm periods are studied at $\mu = 10{,}000$ MeV G^{-1} and $\mu = 13{,}000$ MeV G^{-1} for $K = 0.05\ R_E G^{1/2}$ by analyzing individual passes of the Van Allen Probes (Supplementary Figure 23). For both values of μ, phase space density peaks are again observed, which grow in time during each of the storm intervals, a signature of local acceleration. Our results therefore indicate that heating of relativistic and ultra-relativistic electrons occurred via local acceleration at both $K = 0.11\ R_E G^{1/2}$ and $K = 0.05\ R_E G^{1/2}$, resulting in enhancements at 7 MeV.

The profiles of phase space density at different μ, for two values of K have been considered in this study. The authors highlight that the format of Fig 4 may also be used to layer different values of K at the same μ to compare and contrast how phase space density features appear across the K invariant. This may be a particularly useful tool to explore pitch angle dependent loss mechanisms.

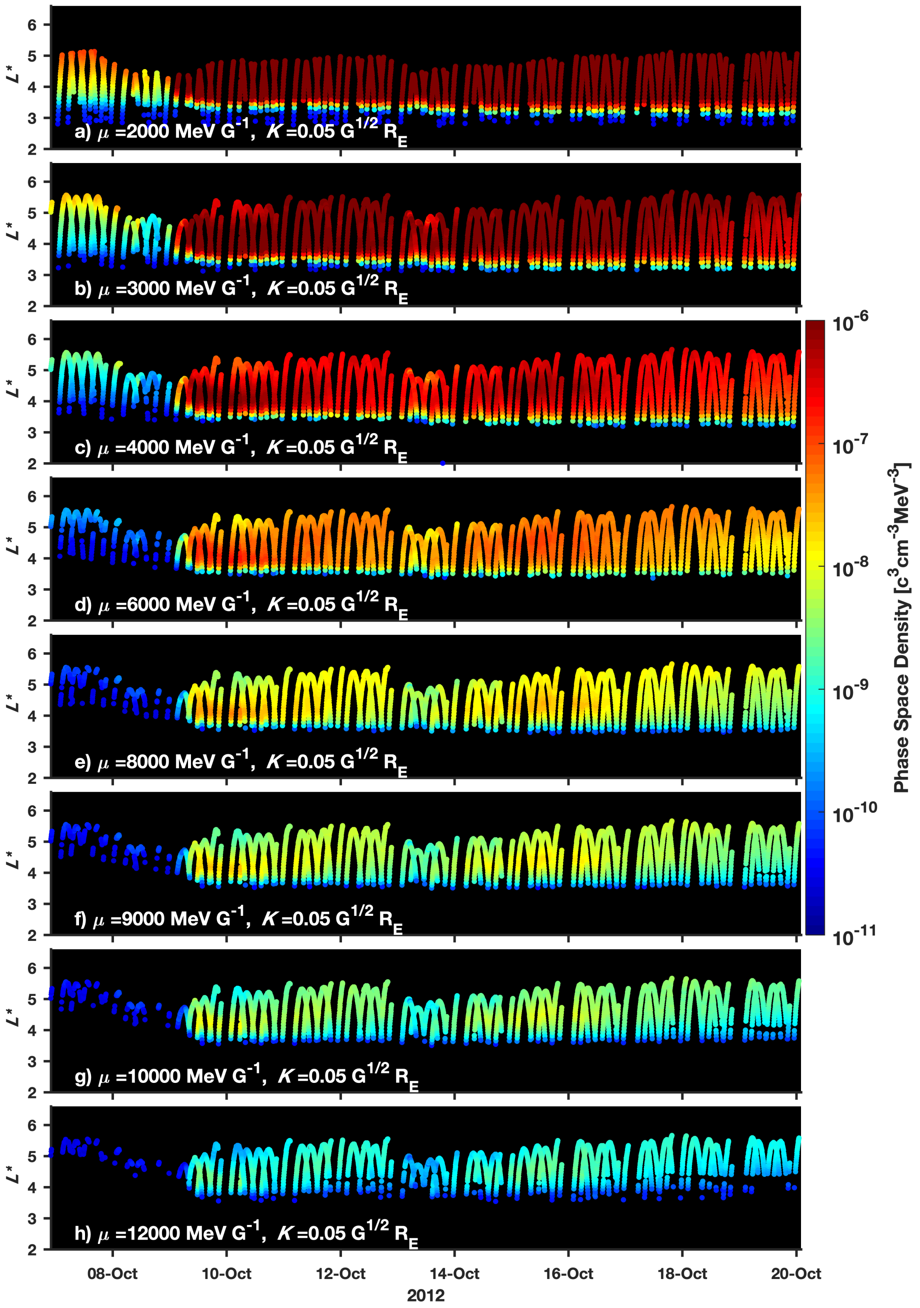

Supplementary Figure 21. Phase space density data throughout orbits of Van Allen Probe A and B. The phase space density at $K = 0.05\ R_E G^{1/2}$ and eight different values of μ (panels a-h) are shown. The figure takes the same format as Supplementary Figure 2. Measurements below the background threshold of the energy channel [6] are not included here. The L^* and K values have been calculated using the TS07 magnetic field model [2], and μ calculated with the locally measured field.

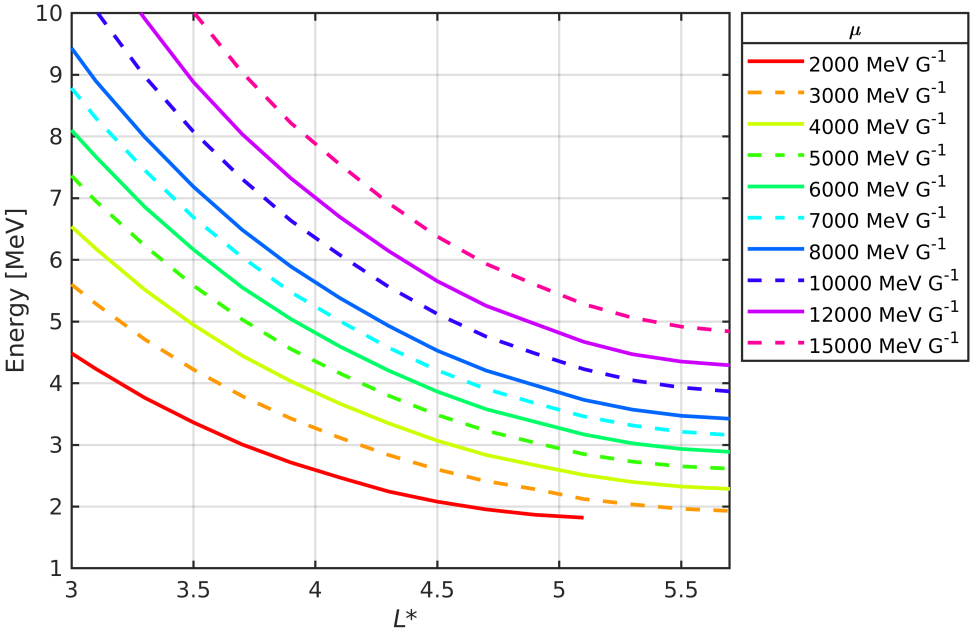

Supplementary Figure 22. Average electron energies at different L^* for $K = 0.05\ R_E G^{1/2}$. Average energies corresponding to μ values between 2,000 and 15,000 MeV G^{-1}, for the 6-20 October 2012 period are shown. The figure takes the same form as Fig 2 in the main text.

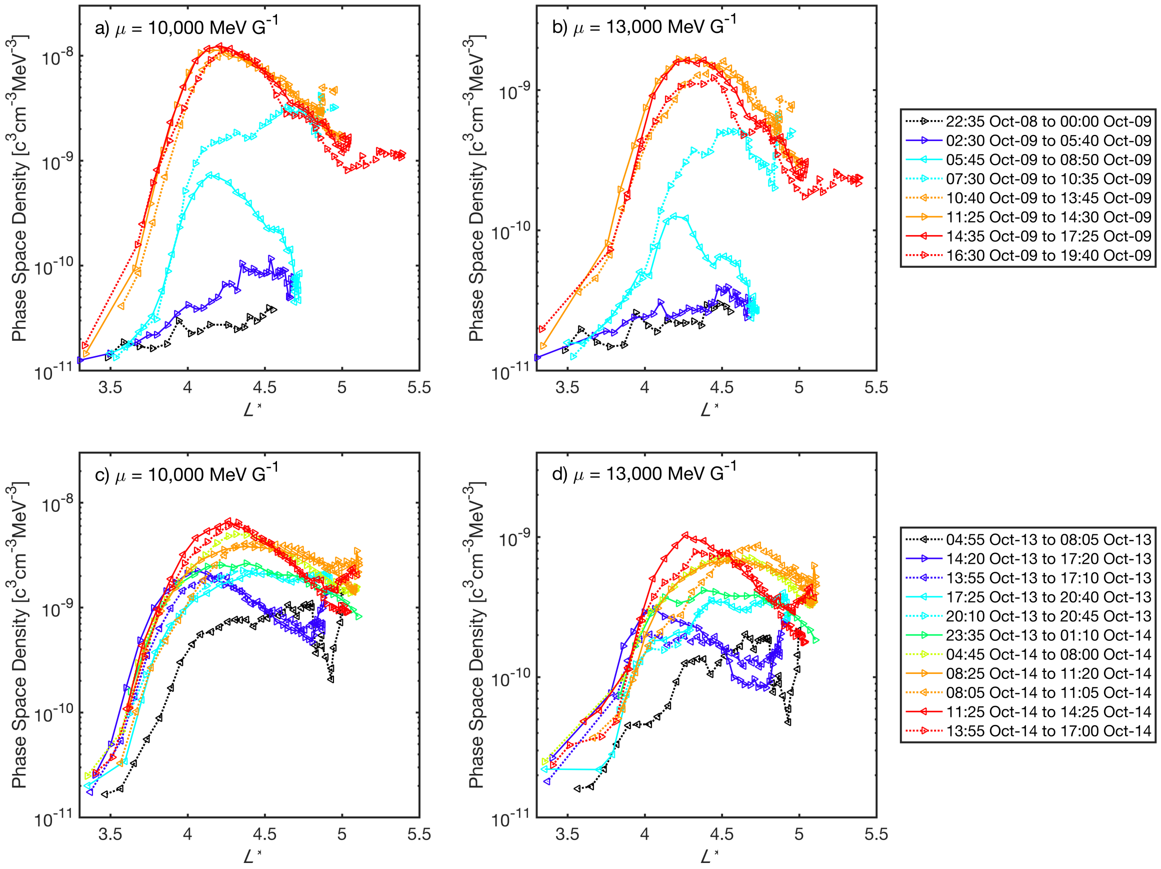

Supplementary Figure 23. Phase space density - L^* profiles for $K = 0.05\ R_E G^{1/2}$. Results from the first storm event are shown for μ = 10,000 MeV G^{-1} and and μ = 13,000 MeV G^{-1} in panels (a) and (b) respectively. Panels (c) and (d) show the phase space density at μ = 10,000 MeV G^{-1} and and μ = 13,000 MeV G^{-1} during the second storm. Van Allen Probe A values are plotted as solid lines and Van Allen Probe B measurements as dotted lines. Arrows indicate the direction of travel.

Supplementary Note 9

Whilst the values of μ shown in this study were calculated using the locally measured magnetic field strength, calculation of K and L^* requires knowledge of the large-scale magnetic field, and therefore a magnetic field model must be used. In the main paper the TS07 model [2] is utilized for these invariants. However, as phase space density is presented in terms of μ, K, and L^*, the profiles observed may change with the choice of magnetic field model. For added rigor, the same analysis is therefore performed using both TS04 and T89.

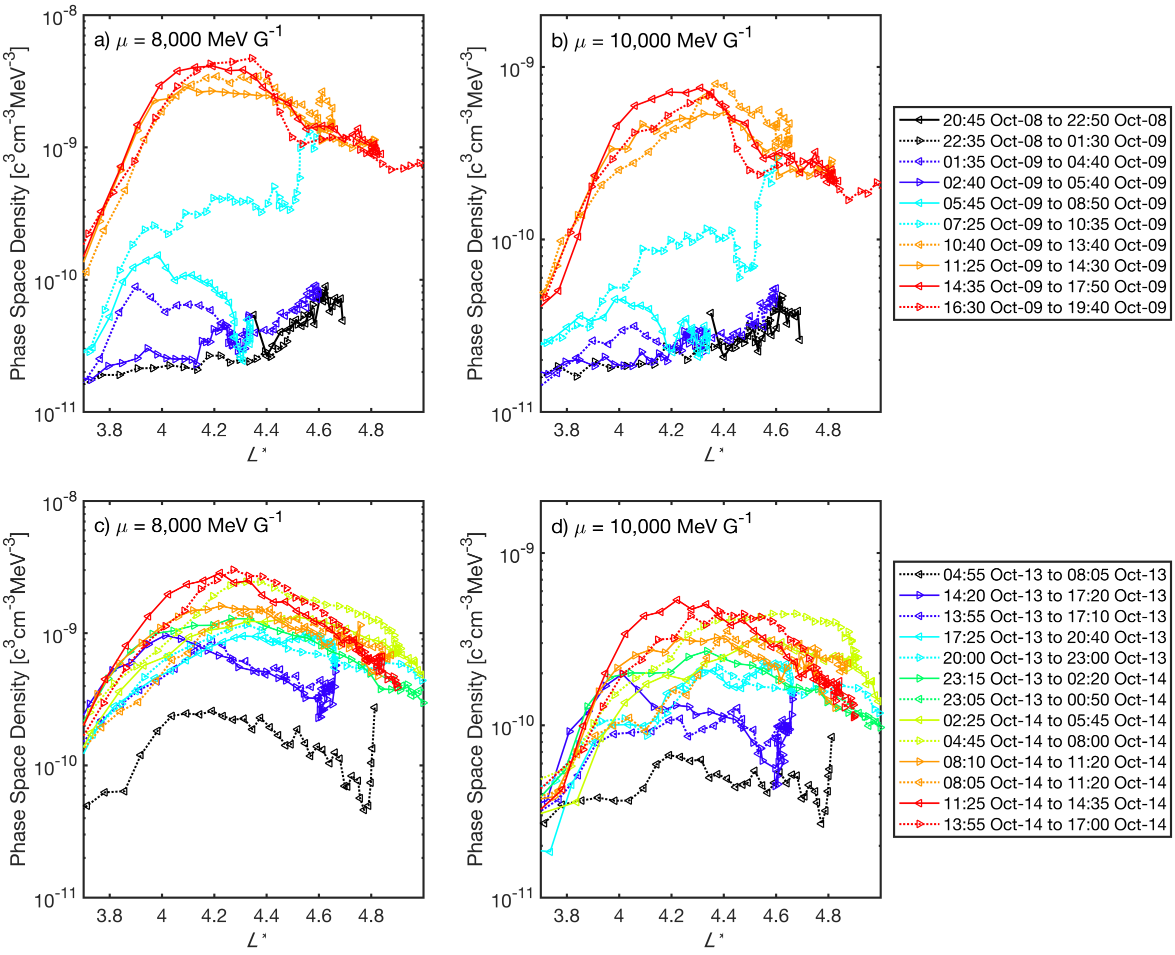

Supplementary Figure 24. Phase space density - L^* profiles with L^* and K from the TS04 field model. μ values are calculated using the locally measured field. Panels (a) and (b) show the phase space density at $\mu = 8{,}000$ MeV G^{-1} and and $\mu = 10{,}000$ MeV G^{-1} during the first storm. Panels (c) and (d) show the phase space density at $\mu = 8{,}000$ MeV G^{-1} and and $\mu = 10{,}000$ MeV G^{-1} during the second storm. Van Allen Probe A values are plotted as solid lines and Van Allen Probe B measurements as dotted lines. Arrows indicate inbound and outbound passes. All panels correspond to $K = 0.11\ R_E G^{1/2}$.

The TS04 external magnetic field model [4] is an empirical model that uses geomagnetic and solar wind parameters to compute parameterization functions. While a predecessor to TS07, this magnetic field model has been used in a number of studies and offers good agreement with observations [17].

Supplementary Figure 24 shows very similar phase space density profiles to when TS07 was used, with growing peaks observed for all three values of μ, during the first and second ultra-relativistic electron enhancement. Using TS04 for the K and L^* calculation, instead of TS07, has not altered the implication of local acceleration up to $\sim$7 MeV.

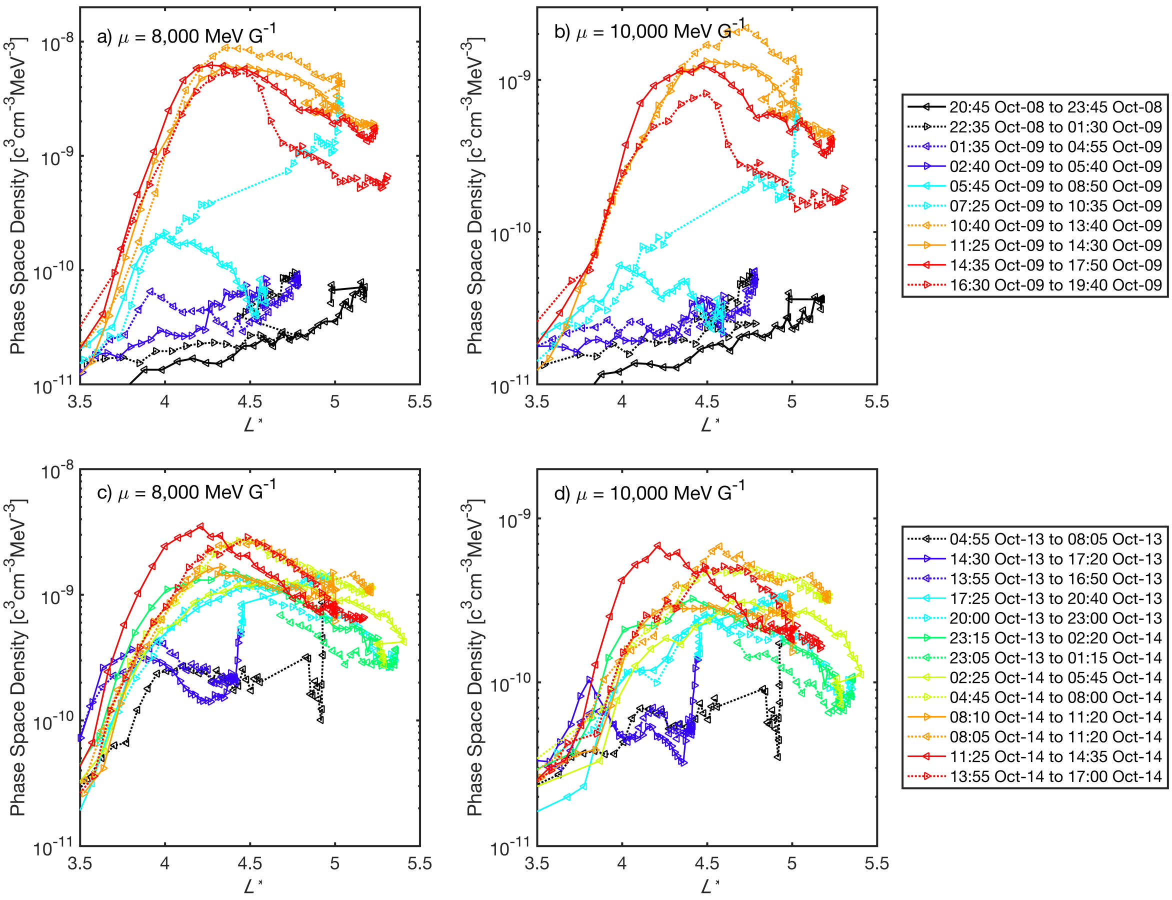

Supplementary Figure 25. Phase space density - L^* profiles with L^* and K from the T89 field model. μ values are calculated using the locally measured field. Panels (a) and (b) show the phase space density at μ = 8,000 MeV G^{-1} and and μ = 10,000 MeV G^{-1} during the first storm. Panels (c) and (d) show the phase space density at μ = 8,000 MeV G^{-1} and and μ = 10,000 MeV G^{-1} during the second storm. Van Allen Probe A values are plotted as solid lines and Van Allen Probe B measurements as dotted lines. Arrows indicate inbound and outbound passes. All panels correspond to $K = 0.11\ R_E G^{1/2}$.

The T89 external magnetic field model [5] is more simplistic than TS07 or TS04, parameterized solely by the three hourly Kp index, but has been widely used for the calculation of both L^* and K [18, 19]. We therefore also consider the phase space density profiles using K and L^* values calculated with T89. Note that, since the Kp index has discrete values, this model introduces some discontinuities in the L^* calculation (as can be seen in Supplementary Figure 25 for the orbit labeled 07:25 - 10:35 09-Oct). In Supplementary Figure 25, using T89, growing peaks are again observed in the phase space density profiles for both the double-dip storm and the second October 2012 storm.

As growing peaks were observed using three different field models, we consider the conclusion that 'local acceleration is largely responsible for the ultra-relativistic electron generation during both storms in October 2012' to be an independent of the selection of TS07.

Supplementary Discussion

Acceleration by inwards radial diffusion is an important process in the radiation belts and will have, in part, contributed to the observed enhancements. Although, for these two storms, the phase space density analysis indicates that it is not the dominant acceleration process. Whenever there are peaks in the phase space density, both inwards and outwards diffusion act [20], but the rate of diffusion in the two directions depends on L^* as well as on gradients in the radial profile. Therefore, in Fig 3, when the combination of local acceleration and either inwards or outwards diffusion is shown schematically, we are referring to the direction where the diffusion is fastest. However, in both instances, outwards and inwards diffusion will occur.

The results presented in this paper do not dismiss inwards diffusion as an important energization mechanism, but rather demonstrate that local acceleration is able to actively heat electrons to 7 MeV.

Whilst phase space density values are subject to uncertainty originating from the required interpolation, the values of μ and K studied, and the magnetic field model used; we have demonstrated growing phase space density peaks for two different values of K, using three different magnetic field models, over a broad range of μ. We therefore conclude that local acceleration mechanisms were active during both of the storms in October 2012 and had a significant affect on the radiation belt particles. Importantly, we have demonstrated that signatures of local acceleration are apparent for electrons with energies up to 7 MeV, suggesting that local heating is responsible for the generation of these ultra-relativistic populations. A combination of local acceleration and inward radial diffusion [21], as a multi-step acceleration process, is therefore not a requirement to form 7 MeV electrons.

Supplementary References

[1] J. G. Roederer. *Dynamics of Geomagnetically Trapped Radiation*, Volume 2. Springer-Verlag Berlin Heidelberg, New York, 1 Edition, (1970).

[2] N. A. Tsyganenko and M. I. Sitnov. Magnetospheric configurations from a high-resolution data-based magnetic field model. *Journal of Geophysical Research: Space Physics*, 112, A06225, (2007).

[3] D. N. Baker, S. G. Kanekal, V. C. Hoxie, S. Batiste, M. Bolton, X. Li, S. R. Elkington, S. Monk, R. Reukauf, S. Steg, J. Westfall, C. Belting, B. Bolton, D. Braun, B. Cervelli, K. Hubbell, M. Kien, S. Knappmiller, S. Wade, B. Lamprecht, K. Stevens, J. Wallace, A. Yehle, H. E. Spence, and R. Friedel. The relativistic electron-proton telescope (REPT) instrument on board the radiation belt storm probes (RBSP) spacecraft: Characterization of Earth's radiation belt high-energy particle populations. *Space Science Reviews*, 179, 337–381, Nov (2013).

[4] N. A. Tsyganenko and M. I. Sitnov. Modeling the dynamics of the inner magnetosphere during strong geomagnetic storms. *Journal of Geophysical Research: Space Physics*, 110, A03208, (2005).

[5] N.A. Tsyganenko. A magnetospheric magnetic field model with a warped tail current sheet. *Planetary and Space Science*, 37, 5 – 20, (1989).

[6] Yuri Y. Shprits, Richard B. Horne, Adam C. Kellerman, and Alexander Y. Drozdov. The dynamics of Van Allen Belts revisited. *Nature Physics*, 14, 102–103, (2018).

[7] Yue Chen, Geoffrey D. Reeves, and Reiner H. W. Friedel. The energization of relativistic electrons in the outer Van Allen radiation belt. *Nature Physics*, 3, 614 – 617, (2007).

[8] Drew L. Turner, Yuri Shprits, Michael Hartinger, and Vassilis Angelopoulos. Explaining sudden losses of outer radiation belt electrons during geomagnetic storms. *Nature Physics*,

8, 208–212, (2012).

[9] C. A. Kletzing, W. S. Kurth, M. Acuna, R. J. MacDowall, R. B. Torbert, T. Averkamp, D. Bodet, S. R. Bounds, M. Chutter, J. Connerney, D. Crawford, J. S. Dolan, R. Dvorsky, G. B. Hospodarsky, J. Howard, V. Jordanova, R. A. Johnson, D. L. Kirchner, B. Mokrzycki, G. Needell, J. Odom, D. Mark, R. Pfaff, J. R. Phillips, C. W. Piker, S. L. Remington, D. Rowland, O. Santolik, R. Schnurr, D. Sheppard, C. W. Smith, R. M. Thorne, and J. Tyler. The electric and magnetic field instrument suite and integrated science (EMFISIS) on RBSP. *Space Science Reviews*, 179, 127–181, (2013).

[10] Y. Y. Shprits, D. Subbotin, and B. Ni. Evolution of electron fluxes in the outer radiation belt computed with the VERB code. *Journal of Geophysical Research: Space Physics*, 114, A11209, (2009).

[11] Yuri Y. Shprits and Richard M. Thorne. Time dependent radial diffusion modeling of relativistic electrons with realistic loss rates. *Geophysical Research Letters*, 31, L08805, (2004).

[12] A. Y. Drozdov, Y Y. Shprits, N. A Aseev, A. C. Kellerman, and G. D. Reeves. Dependence of radiation belt simulations to assumed radial diffusion rates tested for two empirical models of radial transport *Space Weather*, 15, 150– 162 (2017).

[13] D. H. Brautigam and J. M. Albert. Radial diffusion analysis of outer radiation belt electrons during the October 9, 1990, magnetic storm. *Journal of Geophysical Research: Space Physics*, 105, 291–309, (2000).

[14] Nigel P. Meredith, Richard B. Horne, Richard M. Thorne, Danny Summers, and Roger R. Anderson. Substorm dependence of plasmaspheric hiss. *Journal of Geophysical Research: Space Physics*, 109, A06209, (2004).

[15] Richard B. Horne, Richard M. Thorne, Sarah A. Glauert, Jay M. Albert, Nigel P. Meredith, and Roger R. Anderson. Timescale for radiation belt electron acceleration by whistler mode chorus waves. *Journal of Geophysical Research: Space Physics*, 110, A03225, (2005).

[16] A. J. Boyd, D. L. Turner, G. D. Reeves, H. E. Spence, D. N. Baker, and J. B. Blake. What causes radiation belt enhancements: A survey of the Van Allen Probes era. *Geophysical Research Letters*, 45, 5253–5259, (2018).

[17] Chia-Lin Huang, Harlan E. Spence, Howard J. Singer, and Nikolai A. Tsyganenko. A quantitative assessment of empirical magnetic field models at geosynchronous orbit during magnetic storms. *Journal of Geophysical Research: Space Physics*, 113, A04208, (2008).

[18] W. Li, Q. Ma, R. M. Thorne, J. Bortnik, X.-J. Zhang, J. Li, D. N. Baker, G. D. Reeves, H. E. Spence, C. A. Kletzing, W. S. Kurth, G. B. Hospodarsky, J. B. Blake, J. F. Fennell, S. G. Kanekal, V. Angelopoulos, J. C. Green, and J. Goldstein. Radiation belt electron acceleration during the 17 March 2015 geomagnetic storm: Observations and simulations. *Journal of Geophysical Research: Space Physics*, 121, 5520–5536, (2016).

[19] Y. Chen, R. H. W. Friedel, and G. D. Reeves. Phase space density distributions of energetic electrons in the outer radiation belt during two geospace environment modeling inner magnetosphere/storms selected storms. *Journal of Geophysical Research: Space Physics*, 111, A11S04, (2006).

[20] Janet C. Green and M. G. Kivelson. Relativistic electrons in the outer radiation belt: Differentiating between acceleration mechanisms. *Journal of Geophysical Research: Space Physics*, 109, A03213, (2004).

[21] Ch. Katsavrias, I. Sandberg, W. Li, O. Podladchikova, I.A. Daglis, C. Papadimitriou, C. Tsironis, and S. Aminalragia-Giamini. Highly relativistic electron flux enhancement during the weak geomagnetic storm of April–May 2017. *Journal of Geophysical Research: Space Physics*, 124, 4402 – 4413, (2019).
